# Radiation Treatment for Inoperable Local Relapse of Parathyroid Carcinoma With Symptomatic Hypercalcemia: A Case Report

**DOI:** 10.3389/fonc.2021.733772

**Published:** 2021-11-25

**Authors:** Heleen Bollen, Brigitte Decallonne, Sandra Nuyts

**Affiliations:** ^1^ Laboratory of Experimental Radiotherapy, Department of Oncology, University Hospitals Leuven, Leuven, Belgium; ^2^ Department of Radiation Oncology, Leuven Cancer Institute, University Hospitals Leuven, Leuven, Belgium; ^3^ Department of Endocrinology, University Hospitals Leuven, Leuven, Belgium

**Keywords:** case report, parathyroid carcinoma, radiotherapy, radiosensitivity, symptomatic treatment, hypercalcemia

## Abstract

**Background:**

Parathyroid carcinoma (PC) is an extremely rare malignancy, characterized by slow progression, frequent recurrences and difficult-to-control hypercalcemia which is typically the main contributor to the morbidity and mortality of these patients. Patients often undergo repeated surgical resections, whether or not in combination with adjuvant radiation treatment. The role of radiation therapy within the symptomatic treatment of PC currently remains unclear.

**Case description:**

We describe a 30-year-old male patient with an inoperable local relapse of PC and secondary symptomatic hypercalcemia, maximally pharmacologically treated. After a local radiation treatment to a total dose of 70 Gray in 35 fractions serum calcium and parathyroid hormone (PTH) levels decreased, accompanied by improvement of the severe gastro-intestinal disturbances.

**Conclusion:**

For patients with inoperable symptomatic PC despite maximal medical treatment who are in a good overall condition, radiation treatment can be considered in well-defined cases to decrease symptoms and improve quality of life.

## Introduction

Parathyroid carcinoma (PC) represents one of the most rare malignancies. In 1909, de Quervain was the first to report a patient with a PC (1). PC accounts for approximately 11 cases per 10 million people in the United States and less than 1% of all patients presenting with primary hyperparathyroidism (2–8), although a higher proportion has been reported in Asian populations (9). In Belgium, only 20 cases were reported between 2001 and 2010 (10). PC does not have the gender predilection as observed with benign parathyroid tumors, which show a definite female preponderance (3:1) (11). Most cancers present in patients aged between 44 and 60 years (10, 12–16), but a patient as young as 8 years has been reported (17). Clinical features are mostly due to the effects of the excessive secretion of parathyroid hormone (PTH) by the functioning tumor with secondary hypercalcemia, rather than to the tumor burden. The disease is usually diagnosed in an advanced stage, given the non-specific symptoms, and usually has a slow progressive course. Most patients will die due to complications of hypercalcemia, rather than direct tumor invasion or metastases (18–20).

The main treatment for PC is surgery. Given that a complete excision is often technically difficult as the disease is usually diagnosed in a locally advanced stage, persistent or recurrent disease occurs in over 50% of patients (2, 20–22). Patients thus frequently suffer from multiple recurrences, mostly presenting with a gradual rise in PTH and calcium levels, for which numerous surgical resections are often needed. Systemic treatment with the calcimimetic cinacalcet can reduce calcium and PTH levels, but usually loses efficacy over time (12). Cytotoxic chemotherapy has not been proven to affect disease-free or overall survival (13). Since treatment options beyond surgical resection are limited, a radiation treatment (RT) could serve as a valid treatment alternative when surgery of the primary tumor or metastases is no longer feasible. However, PC is generally considered a radio-resistant tumor and literature about RT is limited to the assessment of the value of RT in the adjuvant setting. The rarity of PC has precluded any prospective study and current knowledge about PC is the result of individual case reports and retrospective studies. The role of RT within the symptomatic treatment of PC has not yet been evaluated. We describe a case report of a 30-year-old patient, treated with RT for an inoperable local relapse of PC and secondary symptomatic hypercalcemia. To our knowledge, this is the first case report of symptomatic RT of inoperable PC.

## Case Description

### Patient Information, Clinical Findings, and Diagnostic Assessment

**Figure 1:**
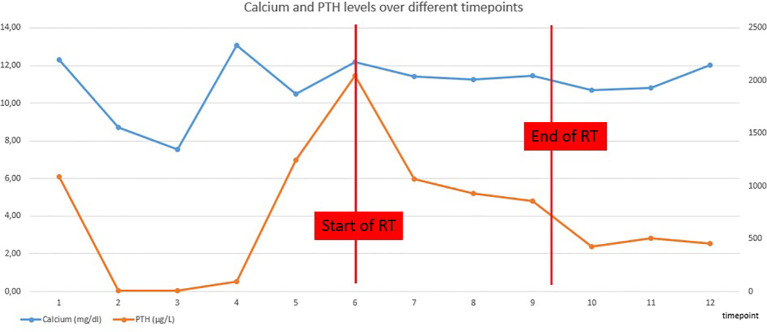
Evolution of serum calcium and PTH concentrations.

A 30-year-old male patient was diagnosed with a PC in 2013, for which he underwent a resection. During the following years, the patient suffered several local relapses, for which 5 more procedures were performed, including local resections, a total thyroidectomy and both a central and bilateral neck dissection. In 2019, the patient presented for the first time in our hospital with persisting PTH-mediated hypercalcemia, while under maximally tolerated dose of the calcimimetic cinacalcet (90mg twice daily) and bone-antiresorptive therapy with denosumab (120 mg once monthly). CT scan showed two suspicious retrosternal lesions, a paratracheal node and a possible adenopathy in lymph node level III at the right side. All suspect locations were resected and a thymectomy was performed. Pathology confirmed the presence of PC retrosternally, in level III on the right, and retroclavicular and paratracheal on the left. As shown in **Table 1** and **Figure 1**, serum calcium and PTH levels decreased after the surgical procedure, and cinacelet and denosumab could be stopped.

**Table 1:** Timeline with evolution of biochemistry, clinical symptoms and radiological features.

	Calcium (mg/dl)	PTH (µg/L)	Phosphate (mmol/l)	1,25 di(OH)-vit D (ng/L)	Clinical and radiological information
**Reference range**	**8.6 - 10.3**	**14.9 - 56.9**	**0.81 - 1.45**	**20.0 - 80.0**	
**Diagnosis local relapse (D0)**	12.30	1086	0.24	143.6	Diagnosis of local relapse, planning of surgical resection. Increase of calcium and PTH under maximal dose of cinacalcet and denosumab.
**D0 + 1 week**	8.70	5	0.96	N.A.	One week after surgical resection of local relapse.
**D0 + 6 weeks**	7.54	7.7	1.81	23.0	Six weeks after surgical resection of local relapse, stop cinacalcet and denosumab.
**D0 + 8 months**	13.07	96	0.54	49.4	Eight months after surgical resection. Increase of calcium and PTH. Restart cinacalcet and denosumab.
**D0 + 12 months**	10.50	1245	0.42	N.A.	Continuous increase of PTH under maximal dose of cinacalcet and denosumab. Diagnosis of inoperable local relapse.
**Start RT (D0RT)**	12.18	2049	0.43	232.0	Referral to RT department. Patient suffers from severe nausea.
**D0RT + 3weeks**	11.42	1069	0.37	N.A.	Three weeks after start of RT.
**D0RT + 4 weeks**	11.26	926	0.28	218.9	Four weeks after start of RT. Decrease of calcium and PTH.
**D0RT + 5 weeks**	11.46	859	0.34	221.5	Five weeks after start of RT. Further decrease of calcium and PTH. Decrease of nausea.
**D0RT + 2 months**	10.70	429	0.44	165.3	Two months after end of RT. Further decrease of calcium and PTH, CT scan shows pseudo-progression.
**D0RT + 7 months**	10.82	504	0.39	204.2	Seven months after end of RT. Calcium and PTH stable, CT scan shows volume decrease.
**D0RT + 9 months**	12.02	452	0.45	232.3	Volume increase on CT scan, recurrence of nausea.

N.A, not available; RT, radiation treatment; PTH, parathyroid hormone; D0, time of diagnosis of the local relapse and planning of surgery; D0RT, time of start RT. As of D0 + 8 months, patient remained under maximal dose of cinacalcet and denosumab.

**Figure 2:**
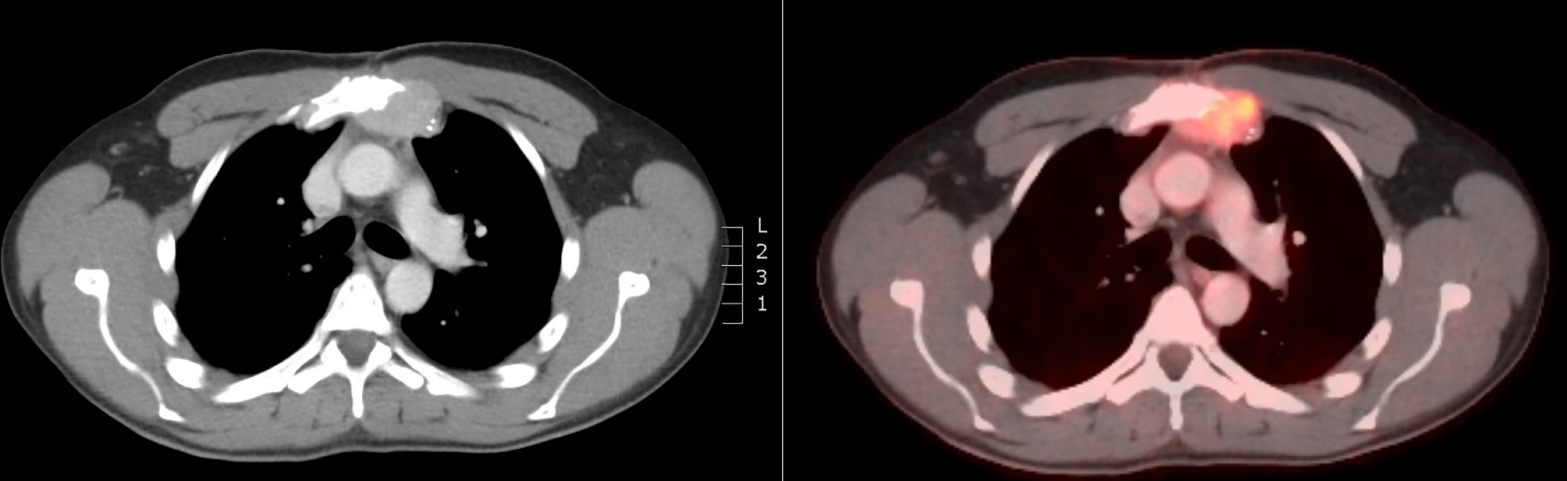
FDG PET/CT at diagnosis of inoperable retrosternal relapse with bone invasion.

Eight months later, the patient experienced a relapse of symptomatic hypercalcemia, in which nausea was predominant, however without convincing tumoral focus at imaging (FDG PET-CT, MRI of the neck, CT of the mediastinum). Cinacalcet was restarted at an intermediate dose. Unfortunately, serum calcium and PTH kept on rising, for which the dose of cinacalcet was increased to maximal dose and denosumab was restarted. With this medical therapy, calcium levels stabilized, remaining at high-normal levels. However, during the following months, serum PTH increased progressively. Repeated imaging now showed a nodular, contrast-capturing lesion between the anonymous vein and the sternum, with erosion of the sternum and the first rib on the left (**Figure 2**).

### Therapeutic Intervention

As the lesion was considered inoperable, the multidisciplinary tumor board decided to opt for a radiation treatment (RT). When patient presented at our RT department, he suffered from severe nausea, which could have been either a side effect from the calcimimetics or directly due to the hypercalcemia. He did not experience pain due to the erosive lesion in the sternum. Based on the few literature that existed, it was decided to deliver a radiation dose of 70 Gray (Gy) in 35 fractions of 2 Gy, using an arc technique with 6 MV photons. Gross Tumor Volume (GTV) was contoured manually and defined as demonstrable macroscopic disease on CT. The Clinical Target Volume (CTV) consisted of a 10-millimetre margin around the GTV, corrected for bone and air cavities. Another 5 millimetres were added for the construction of the Planning Target Volume. Volumetric Modulated Arc Therapy (VMAT) was used, using 2 arcs allowing a precise shaping of the dose to the form of the tumor. Daily cone beam CT imaging was performed to improve the precision and accuracy of the delivery of radiation treatment. Just before the start of RT, the patient developed acute sternal pain, caused by a sternal fracture for which a conservative treatment with analgesics was proposed. The pain disappeared quickly during the RT.

### Follow-Up and Outcomes

During the radiation treatment, the patient reported markedly less gastro-intestinal discomfort. At the end of the treatment, he did not need any symptomatic anti-nausea medication and there was significant weight gain. Furthermore, there was a good tolerance for the treatment: patient developed a grade I dermatitis, there was no dysphagia. Serum calcium and PTH levels were checked weekly during RT and showed a progressive decline (**Table 1** and **Figure 1**).

**Figure 3:**
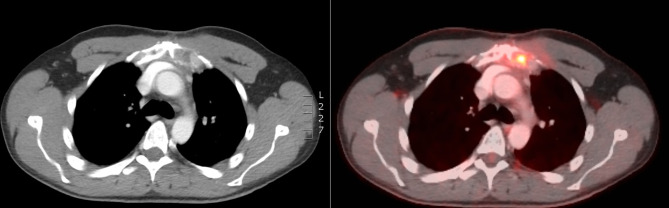
CT scan at 2 months after RT showing volume increase of the sternal lesion, probably due to pseudo-progression.

**Figure 4:**
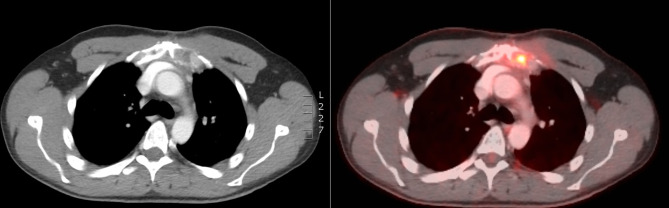
CT scan 7 months after the end of RT showing volume decrease of the sternal lesion.

Two months after the end of the RT, the patient was still free from nausea and PTH and calcium levels were progressively decreasing. On CT-scan, a small volume increase of the sternal lesion was described. This increase was presumably due to pseudo-progression resulting from tumor necrosis, based on the density of the lesion and decreasing levels of calcium and PTH (**Figure 3**). Five months later, the clinical and biochemical situation was stable and CT-scan showed a volume decrease of the sternal lesion (**Figure 4**). The patient did not experience any pain and nausea was controlled under maximal dose of calcimimetics and bone antiresorptive therapy. Unfortunately, two months later, he experienced a return of gastro-intestinal disturbances. An increase of serum calcium levels was observed and a volume increase of the sternal lesion was confirmed on FDG PET-CT. Patient was referred for inclusion in a medical trial, in which he is currently treated with PARP (poly ADP ribose polymerase)-inhibition.

## Discussion

In this report, we describe a case of a patient with an inoperable sternal relapse of PC with disabling nausea secondary to malignant PTH-mediated hypercalcemia. Upon RT, which was well-tolerated, the patient reported a significant improvement of the gastro-intestinal discomfort for seven months, during which anti-nausea medication could be stopped and body weight gain occurred. This clinical evolution was paralleled by a decline of PTH and calcium levels until two months after RT, and calcium and PTH levels remained stable for 7 months after RT.

### Prognosis of PC

The only series reporting survival in PC patients are those in which surgery was the primary treatment modality. The M. D. Anderson series of 27 patients showed a 5-year overall survival (OS) of 85% and a 10-year OS of 77% (22). The authors reported no significant association between any demographic or pathologic feature and prognosis. A recent National Cancer Database Analysis on 885 patients reported a 5-year and 10-year OS of 85.4% (95% CI 82.4-87.9%) and 67.1% (95% CI 61.7-72.0%) (3).

### Surgical and Pharmacological Treatment

The cornerstone of the treatment for PC is surgery. Complete surgical en bloc resection with ipsilateral hemithyroidectomy and prophylactic central lymphadenectomy (level VI) is generally recommended, and microscopic negative margins are considered the best chance for cure (2, 3, 20, 23–26). Lymphadenectomy of regional lymph nodes other than level VI is not recommended as no therapeutic benefit has been established (22, 27). The majority of patients is only diagnosed with PC after surgery, which means that most resections are incomplete (21). Early surgical re-excision is recommended in patients who are diagnosed after simple parathyroidectomy (14, 15). Even in the case of a curative resection, PC has a recurrence rate of more than 50% (2, 20–22). Most recurrences occur 2–3 years after the initial operation, but this period is variable and a prolonged disease-free interval of as long as 23 years has been reported in the literature (2, 21). This emphasizes the importance of long-term follow-up of patients after surgery. A short disease-free interval is associated with poor prognosis. Recurrent disease mostly presents with rising levels of serum calcium and PTH. Surgery is the most effective treatment for recurrent PC (21, 28, 29). Reoperation has been proven to decrease PTH and calcium levels and to improve symptoms and is thus recommended when feasible (12, 30). Unfortunately, reoperations for parathyroid cancer are rarely if ever curative (12, 27, 31).

Most PCs are functional, where only a few are non-functional with normal serum PTH and calcium levels (12). Although there are no biochemical or radiologic diagnostic criteria for PC, serum calcium levels are generally higher than in parathyroid adenomas (32, 33). Symptoms and signs of PC are mostly secondary to hypercalcemia rather than expansion of the tumor itself. The most frequent complaints are nausea, anorexia, vomiting, weight loss, dyspepsia, fatigue, constipation, headaches, myopathy, neurocognitive deficits, polydipsia and polyuria. Bone, joint, muscular pain, pathological fractures and renal stones are also frequent. As patients usually die from the metabolic complications of hypercalcemia, medical treatment with calcimimetics and bone-antiresoptive drugs and if needed forced hydratation with loop diuretics is indicated for long-term control of hypercalcemia (11, 21, 34). Although usually partially effective, medical therapy often loses efficacy over time (12).

In this particular case, the question arose as to the best approach, given that a surgical operation was no longer possible and the patient suffered from severe symptomatic hypercalcemia, despite maximal medical treatment with calcimimetics and bone antiresorptive therapy.

### The Role of Radiotherapy in the Treatment of PC

As the treatment of PC is mainly surgical, literature about RT is limited to contradictory studies, evaluating the benefits of adjuvant RT. To our knowledge, no trials or case reports exist regarding RT without prior surgery as treatment of PC. PC is considered radio-resistant and therefore adjuvant RT has traditionally not been deemed effective (6, 14, 20, 26, 35–37). However, a few small case series have demonstrated lower recurrence and longer disease free survival with the use of adjuvant RT, although without significant overall survival benefit (2, 18, 22, 34, 38–40). The Mayo Clinic has reported a disease-free survival at a median follow-up period of 60 months in 4 patients who received postoperative RT (34). The M.D. Anderson experience suggests a lower local recurrence rate if adjuvant radiation is given after surgery, independent of the type of operation and the disease stage (40). These studies provide some evidence that PC may be a radiosensitive tumor and adjuvant RT may have a role in the control of locoregional disease progression (11, 41). A recent large National Cancer Database Analysis did, however, not show any survival benefit of RT in the adjuvant treatment of PC (3). All trials dealing with RT as adjuvant treatment include a small number of patients without any comparison, thus no strong conclusion can be drawn. Based on the existing data, the choice was made not to deliver adjuvant RT after surgical resection of the retrosternal relapse.

Literature does not provide an answer about the role of RT when systemic therapy proves insufficient as symptomatic treatment. The rarity of PC renders a randomized prospective trial very difficult, hindering the generation of sufficient statistical power through a large number of patients. Guidelines published by the American Association of Endocrine Surgeons (AAES) state that RT can be considered in patients with refractory disease who are not candidates for re-operation (23). As our patient met these conditions, the multidisciplinary team decided to deliver RT to a total dose of 70 Gy in 35 fractions of 2 Gy. Regarding the decision about the delivered radiotherapy dose, the existing literature is limited and only addresses the issue of the radiation dose in an adjuvant setting. Furthermore, results are statistically underpowered and little information is provided about the extent of surgical resection. In both Chow et al. and Christakis et al., all 10 patients underwent the standard resection with adjuvant RT up to 40 Gy. No recurrences were identified over a follow-up of 1 to over 12 years (18, 38). Doses of 50 to 66 Gy in adjuvant setting are reported by M.D. Anderson and Mayo Clinic (22, 34). We relied upon our experience with RT in rare cases of thyroid cancer, such as anaplastic thyroid cancer, and decided to deliver a curative dose of 70 Gy since the patient was young and in good physical condition. Our patient experienced a continuous decrease of calcium and PTH levels during two months after the RT, which was translated into a significant reduction of gastro-intestinal complaints. Both metabolic symptoms and biochemical values remained stable up until 7 months after RT. The result of the RT is promising and clinically relevant since further treatment options were limited and the patient’s quality of life improved for a significant period of time. Furthermore, a clear tumor volume decrease was observed on a CT scan performed 7 months after the end of treatment. Together, these findings suggest radiosensitivity of PC, with a reduction of both tumor load and metabolic consequences.

Trials or case reports regarding a symptomatic radiation treatment of PC to decrease metabolic complaints are scarce and it is difficult to draw conclusions from the literature that is available. A decrease of PTH levels during adjuvant RT has been reported (19). Some older studies, performed on patients with bulky neck disease who had not undergone surgery, failed to demonstrate either a reduction of tumoral mass or the attainment of normocalcemia with RT (38, 42). One case report mentions beneficial biochemical and symptomatic effects of radiofrequency ablation of 10 liver metastases of parathyroid carcinoma in a 71-year-old patient (43). The latter suggests a benefit of a radical treatment of either a local relapse or metastatic disease, which supports our choice for a curative dose of 70 Gy.

Since our patient was very young, had a good overall condition and was not responding any longer to calcimimetics and bone antiresorptive therapy, we considered RT as a possible modality to prevent the patient from developing pain due to the local relapse and to reduce metabolic complaints. The experienced side effects of RT were minimal, so the choice for RT seemed to be justifiable in this particular case. However, the development of side effects strongly depends on the location of the relapse, so benefits and drawbacks of RT need to be outweighed for each patient individually. The decision on the most suitable treatment is thus preferably taken by an experienced multidisciplinary tumor board in a high-volume center with extensive experience with head and neck tumors.

Future treatment options, including immunotherapy, multi Kinase and Poly ADP Ribose Polymerase inhibitors, are currently under investigation and, with further refining, will hopefully become a part of the arsenal to treat PC. With regard to RT, particle therapy such as proton and carbon ion therapy might provide the opportunity to provide a higher RT dose to the target volume (TV), while minimizing possible side effects. In this particular case, considering the intrinsic radio-resistance of PC, the proximity of the TV to the lungs and the heart and the young age of the patient, particle therapy could be valuable. However, given the lack of evidence, further research is needed. In the meantime, there may be a role for symptomatic X-ray radiation therapy in well-defined cases.

## Conclusion

PC remains difficult to treat, with limited effective treatment options beyond surgical resection. In inoperable cases, refractory to medical treatment with calcimimetic agents and bone antiresorptive drugs to achieve metabolic and symptomatic control, radiation therapy can be considered. The presented case suggests radiosensitivity, resulting in both a reduction of both tumor load and control of malignant hypercalcemia-related symptoms.

## Data Availability Statement

The original contributions presented in the study are included in the article/supplementary material. Further inquiries can be directed to the corresponding author.

## Ethics Statement

The studies involving human participants were reviewed and approved by the Ethics Committee Research UZ/KU Leuven. The patients/participants provided their written informed consent to participate in this study. Written informed consent was obtained from the individual(s) for the publication of any potentially identifiable images or data included in this article.

## Author Contributions

Writing manuscript draft, HB. Writing review and editing, SN, BD and HB. Supervision, SN and DB. All authors have read and agreed to the published version of the manuscript.

## Conflict of Interest

The authors declare that the research was conducted in the absence of any commercial or financial relationships that could be construed as a potential conflict of interest.

## Publisher’s Note

All claims expressed in this article are solely those of the authors and do not necessarily represent those of their affiliated organizations, or those of the publisher, the editors and the reviewers. Any product that may be evaluated in this article, or claim that may be made by its manufacturer, is not guaranteed or endorsed by the publisher.
